# Vestibular prosthesis: from basic research to clinics

**DOI:** 10.3389/fnint.2023.1161860

**Published:** 2023-05-16

**Authors:** Enrique Soto, Adriana Pliego, Rosario Vega

**Affiliations:** ^1^Benemérita Universidad Autónoma de Puebla, Instituto de Fisiología, Puebla, Mexico; ^2^Universidad Autónoma del Estado de México (UAEMéx), Facultad de Medicina, Toluca, Mexico

**Keywords:** vestibulopathy, vestibular implant, galvanic vestibular stimulation, sensory substitution, balance disorders, vestibular prosthesis

## Abstract

Balance disorders are highly prevalent worldwide, causing substantial disability with high personal and socioeconomic impact. The prognosis in many of these patients is poor, and rehabilitation programs provide little help in many cases. This medical problem can be addressed using microelectronics by combining the highly successful cochlear implant experience to produce a vestibular prosthesis, using the technical advances in micro gyroscopes and micro accelerometers, which are the electronic equivalents of the semicircular canals (SCC) and the otolithic organs. Reaching this technological milestone fostered the possibility of using these electronic devices to substitute the vestibular function, mainly for visual stability and posture, in case of damage to the vestibular endorgans. The development of implantable and non-implantable devices showed diverse outcomes when considering the integrity of the vestibular pathways, the device parameters (current intensity, impedance, and waveform), and the targeted physiological function (balance and gaze). In this review, we will examine the development and testing of various prototypes of the vestibular implant (VI). The insight raised by examining the state-of-the-art vestibular prosthesis will facilitate the development of new device-development strategies and discuss the feasibility of complex combinations of implantable devices for disorders that directly affect balance and motor performance.

## The need for a vestibular prosthesis

The control of posture in bipedal animals involves a complex integration of proprioceptive, vestibular, and visual information (Fife, 2010; Cullen, 2012). The peripheral vestibular organs are the natural sensors of linear and angular accelerations of the head. Sensory input coming from the vestibular organs converges to the vestibular nuclei in the central nervous system (CNS), defining the cephalic position relative to gravity and motion dynamics, integrating input from other sensory systems to produce both voluntary and reflex responses that include vestibulo-spinal, vestibulo-collic, vestibulo-ocular, and vestibulo-autonomic activity (Basci and Colebatch, 2005; Jamon, 2014). In addition, the vestibular system involves superior nervous structures that prevent oscillatory movement, define a vertical reference to realign the body, and contribute to motion perception. Some brain regions that participate in subject stabilization and navigation are the insula in the temporoparietal cortex, the cerebellum, the hippocampus, and the entorhinal cortex (Hitier et al., 2014). The vestibular activity is fundamental for spatial navigation, generating an internal map of inertial reference and cognitive processes related to the body scheme (Lopez, 2013; Alme et al., 2014; Hitier et al., 2014).

The World Health Survey indicates that the prevalence of disability in adults is 2.2%, while data from the Global Burden of Disease Study indicate that 3.8% of the adult population has a “serious disability” (World Health Organization [WHO], 2011). In general, 22.2% of American adults report some disability that affects mobility; the most frequent type is vestibular disorders (13.0%) (Courtney-Long et al., 2015). In South Korea, a survey (2010 to 2012) evaluated 12,653 participants (5,450 men and 7,203 women); the prevalence of dizziness in 1 year was 20.10%. Dizziness was more frequent among women (25.18%) than among men (14.57%; *P* < 0.001), and the prevalence rate increased with age (*P* < 0.001). The associated factors also included high triglycerides, smoking, and alcohol consumption (Chang et al., 2018).

Recent studies have confirmed this data in other countries and shown the prevalence of dizziness and vertigo (with no origin of vertigo specified) in around 15 to 20% of the population. Vestibular vertigo accounts for about a quarter of dizziness complaints, with a prevalence of 5% and an annual incidence of 1.4%. Its prevalence increases with age and is two to three times more frequent in women than men (Lucieer et al., 2016; Neuhauser, 2016; Grill et al., 2018).

According to the Barany Society criteria, “Bilateral vestibulopathy is a chronic vestibular syndrome characterized by unsteadiness when walking or standing, which worsens in darkness and on uneven ground, or during head motion. Additionally, patients may describe the head or body movement-induced blurred vision or oscillopsia. There are typically no symptoms while sitting or lying down under static conditions” (Strupp et al., 2017). In most patients with bilateral vestibular dysfunction (BVD), the central compensatory mechanisms, including sensory substitution and plastic changes in the central nervous system, which may compensate for unilateral vestibular damage, are insufficient; therefore, the prognosis is poor and therapeutic options are limited (McCall and Yates, 2011; Hain et al., 2018).

Experiments in animals have shown that non-labyrinthine inputs to the vestibular nuclei reweighting following a BVD, which may account for the recovery of postural stability and orthostatism. However, vestibulo ocular reflex (VOR) loss and spatial cognition are frequently permanent (McCall and Yates, 2011). Due to its prevalence, high impact on quality of life, and poor prognosis, patients with profound peripheral BVD or bilateral vestibular loss constitute the group of interest for clinical research and rehabilitation with prosthetic devices (Hall et al., 2016). The group with BVD is the most likely to receive an implantable vestibular prosthesis (Strupp et al., 2017). In contrast, those patients with mild vestibular hypofunction, either unilateral (uncompensated), bilateral, or with presbyvestibulopathy, are the leading interest group for non-implantable auxiliary devices.

## Physiology of electrically stimulated vestibular nerve

Cohen and his colleagues did pioneer work in the development of vestibular prostheses. They were the first to study the effects of implanted electrodes to selectively stimulate the SCC and the utricle in cats, rabbits, and monkeys. They found that during ampullary nerve stimulation, the head and eyes moved in planes parallel to the plane of the canal of the stimulated nerve. The induced activity in the ampullary nerves was synchronous with the frequency of electrical stimulation, and eye movements followed pulse trains of up to 100 Hz (Cohen and Suzuki, 1963; Cohen et al., 1964; Suzuki et al., 1969).

The response of vestibular nerve afferents to current stimulation was thoroughly studied in the squirrel monkey (Goldberg et al., 1984). The sensitivity of vestibular afferents to galvanic stimulation was found to be linearly related to the discharge regularity of the neurons, regardless of their origin in the saccule or the otolithic organs. Depending on the geometry between the electrode and nerve axons, anodic direct current extracellular stimulation inhibited neural activity, whereas cathodic current provoked an increase (Goldberg et al., 1982, 1984). In the isolated labyrinth from guitarfish, the electrical polarization of the nerve could induce an increase or a decrease in the afferent neuron discharge. This action was mainly due to an effect of the current on the action potential generator mechanism of afferent neurons (Macadar and Budelli, 1984). These authors also showed that utricular afferent neurons’ response to electrical polarization was analogous to their response to mechanical stimuli, including adaptation to sustained stimuli.

Electrical stimulation efficacy may decrease after long-time use due to electrode polarization and reactive fibrosis in the vicinity of the electrodes. Electrode polarization may be overcome by using brief, charge-balanced cathodic-anodic pulses. However, the cathodic, excitatory phase dominates the neural response, making it challenging to achieve neuronal discharge inhibition. Recently, microfluidics has allowed the design of a direct current stimulator to inject DC inhibitory current at the output of the device (Fridman, 2017). Direct cathodic stimulation paired with pulses modulated the vestibular activity across an operating range better than pulse-modulated frequency stimuli and seems more efficient in modulating the vestibular system in a wide range of head velocities than with pulse-modulated frequency alone (Aplin et al., 2019). Fibrosis and subsequent lack of contact of electrodes may be overcome by immobilized protein coatings or organic coatings of electrodes and the core metallic made typically of platinum (Pt), platinum-iridium (Pt-Ir), and gold. However, many proposals and studies in this field are in progress (For a review, see Lotti et al., 2017).

Critical aspects of the stimulation procedure, such as electrode design, positioning, and characteristics of electrical stimuli (current intensity and waveform), pulse rate and current modulation to an external input (i.e., movement) or both (amplitude and rate modulation), are the subject of intensive research analysis. The ideal waveform (periodic, step signal, or band-limited noise) for effective nerve stimulation is still unclear. Most studies have used DC and square bipolar pulses for nerve stimulation, although some evidence suggests that triangular waveform or noise may be a more efficient stimulus (Schier et al., 2018). Electrode location, intra- or extra-labyrinthine, and the effectiveness due to nerve proximity are being researched.

Our work showed that electrical field stimulation in the isolated vestibule significantly influenced the discharge rate of vestibular afferent neurons. DC stimuli elicited an initial discharge rate increase, followed by an adaptation process that decreased the discharge rate toward a basal level. Sinusoidal and pulse stimulation produced a phase-locked increase in the discharge rate, which showed no adaptation. Notably, a sustained discharge throughout the stimulation period (20 s) was elicited with white noise stimulation, increasing the spike activity, which showed a sigmoidal dependence on the stimulus amplitude (Vega et al., 2022).

Alternative stimulation methods, apart from electrical stimulation, have been evaluated. Infrared light (IR) produced a change in neural activity. The data suggest that pulsed IR activates sensory hair cells, thus leading to the modulation of synaptic transmission and afferent nerve discharge (Rajguru et al., 2011). Further studies in rats showed that IR stimuli produced eye movements that were stable through repeated cycles of stimulation for several hours (Jiang and Rajguru, 2018). Infrared and near-infrared light neuronal stimulation is spatially selective and feasible, as shown in various animal models. It is thought that optical stimulation will enable neural prostheses with enhanced neural fidelity (for a review, see Littlefield and Richter, 2021). The heat generated by water absorption of photons in the infrared band and heating changes the membrane capacitance, resulting in a depolarizing inward current. Vanilloid transient receptor potential channels (TRPV) are the primary effectors of the chain reaction triggered by laser irradiation in vestibular neurons (Albert et al., 2012). The use of ultrasound pulses was also studied. In the toadfish, 5 MHz low-intensity, focused ultrasound activated otolithic organs; the neuronal evoked activity was like that produced by direct mechanical stimulation of the otolith (Iversen et al., 2017). The feasibility of an optogenetic approach has also been discussed for cochlear prosthesis, based on the auditory brainstem response recorded when optogenetically stimulating the spiral ganglion neurons in animal models. However, the translational potential of an optogenetic-based prosthesis is still far from being solved (Dieter et al., 2020).

## Prosthesis design

As previously described, SCC sense angular accelerations and otolithic organs sense linear accelerations (Curthoys et al., 2018; Rabbitt, 2019). In electronics, gyroscopes are instruments for sensing angular acceleration and accelerometers for sensing linear acceleration. In the 1990s, the development of micro-gyroscopes and micro-accelerometers opened the possibility of the development of an artificial vestibule formed by microchips, including triaxial gyroscopes and accelerometers small enough to be used by a human being (Gong and Merfeld, 2002; Liu and Shkel, 2003; Liu et al., 2003; Wall et al., 2002/2003; Alexandrov et al., 2004). Gyroscopes and accelerometers are now so sensitive that they can detect head movements in microgravity (Sadovnichii et al., 2018). It is worth noting that recent advances in biomimetic SCC and otolithic organs development can improve the sensing capability of prosthetic devices, and because of their mechanical behavior, their output sensitivity and frequency selectivity will be analogous to natural systems producing a more reliable operation of prosthetic devices with less computational processing demand (Raoufi et al., 2019; Jiang et al., 2022; Moshizi et al., 2022).

**FIGURE 1:**
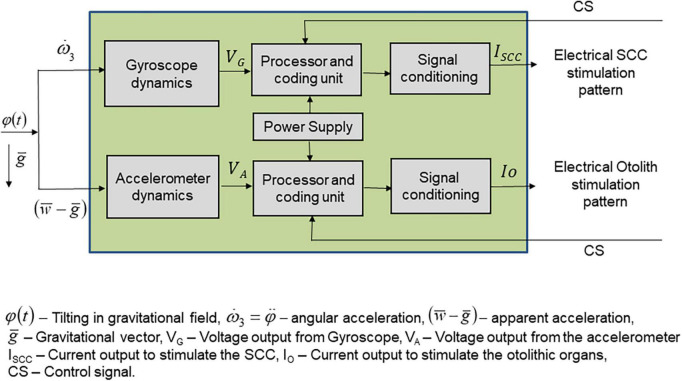
Schematics of the elements constituting a vestibular prosthesis. The input to the prosthetic device is in the form of angular and linear accelerations produced by head movements, within the gravitational field. Sensors are constituted by accelerometers that respond to linear accelerations and gyroscopes which respond to angular accelerations, thus forming two flows of activity to be processed separately. The sensors are in the three spatial planes. The output of the sensors is a voltage, which feeds the processing unit. This unit converts the input voltage in a stimulation pattern defined by a transfer function adequate and differentiated for the linear accelerations-related input and for the angular accelerations input. The processor and coding unit output feeds a signal conditioning unit that converts the stimulation pattern in a current to be applied to the otolithic afferent neurons in one case and to the three semicircular canals in the other case.

Major problems include prosthetic coding for the whole range of head velocities, the development of a transfer function for output to code for the whole range of head velocities, the definition of a stimulus pattern that will overcome the variability and limited response of vestibular nerves to electrical stimulation, and the definition of protocols that may help to improve the selectivity of nerve responses (Figure 1).

Most authors have focused on replacing the semicircular canal function, as reviewed by van de Berg et al. (2011); this is likely because of the simpler surgical approach to SCC ampullae. Also, the function of an SCC device can be analyzed directly by measuring the VOR. Thus, prosthetic devices typically use a sensing unit based on gyroscopes and, only recently also, accelerometers. The lack of an accelerometer in a device implies that no information about the head’s position and translational displacements is provided to the user. Additionally, mathematical modeling has shown that, in a fall, because the head is out of the rotation center of the body, there is a significant linear component, and accelerometers detect the beginning of the fall better than gyroscopes sensors (Sadovnichy et al., 2007). Therefore, it would be ideal to include accelerometers to stimulate otolithic nerves, allowing the subject to detect the stability of the head and translational (linear) displacements to prevent a fall.

The otoliths can also encode head rotations and contribute to angular VOR compensation (Sadovnichy et al., 2007; Ramos de Miguel et al., 2017). However, the topographical organization of otolithic end-organs is complex. For these reasons, the implanted prosthetic devices should provide valuable information to restore basic orientation information and improve the subjects’ quality of life. Using a combination of implanted and non-implanted devices would probably constitute the best option for the immediate future of vestibular prostheses.

An additional aspect of developing a vestibular prosthesis is the concept of a transfer function that converts the sensor output into an adequate stimulation pattern for vestibular afferent neurons. Most authors have used a linear or sigmoidal transfer function to code the gyroscope output as a frequency-modulated output (Gong and Merfeld, 2002; Della Santina et al., 2005, 2007; Töreyin and Bhatti, 2013; Perez-Fornos et al., 2014; Boutros et al., 2016; van de Berg et al., 2017a). A neuromimetic mathematical model to code the gyroscope or accelerometer output into a series of frequency-modulated pulses has been proposed (Sadovnichy et al., 2007; Vega et al., 2008, 2016; Alexandrov et al., 2014). Theoretical analyses using this model reproduce the afferent activity in response to linear or angular accelerations (mechanical coupling differs for both), predicting the complex behavior of afferent neurons, including repetitive spike discharge. In addition, the model predicts that some small-amplitude random noise at the input would improve the system’s gain (McDonnell and Abbott, 2009; Alexandrov et al., 2014, 2015). A novel approach used a high-pass biomimetic mapping to control the vestibular prosthesis based on regular, irregular, and mixed afferent vestibular properties in two implanted rhesus monkeys, showing an improvement in high-frequency VOR gain and better phase compensation compared to a static sigmoidal mapping of head rotation to pulse rate. The performance observed supports the argument that a more “natural coding” behavior could also improve the prosthetic function in human subjects (Wiboonsaksakul et al., 2022).

How much additional complexity of signal processing is adequate to produce effective vestibular responses has not yet been defined; however, it is feasible to imagine that, in the future, ideal devices should combine mathematical modeling and some contextual analyses to get closer to a natural function of vestibular endorgans. An obvious limitation of complex processing is the computer power required to process the sensor’s output. An advantage is that such a system would provide a non-linear adaptive activation of the afferent neurons, which may thus resemble the natural activation of the system.

## Implantable vestibular prosthesis (animal experiments)

The research group at the Jenks Vestibular Physiology Laboratory (Massachusetts Eye and Ear Infirmary, Boston, MA, USA) was the first to develop a detailed proposal for a vestibular prosthetic device tested in animal models. The earliest device measured the angular velocity of the head (±500 degrees/s) using a piezoelectric vibrating gyroscope and a filtered output to modulate stimulus rate between 50 and 250 Hz via a sigmoidal lookup table, which related pulse rate to the angular velocity. The device was mounted on the head of a guinea pig with surgically implanted platinum electrodes. Results showed that current pulse injection produced eye movements modulated by the angular acceleration sensor, providing a rotational cue to the animal (Gong and Merfeld, 2000).

For later testing of this device, branches of the horizontal semicircular canal of the guinea pig were surgically plugged and stimulated using a baseline frequency set at 150 Hz to restore the basal discharge of afferent neurons, allowing excitatory or inhibitory modulation of nerve activity. A gyroscope modulated the pulse rate above or below the baseline frequency. After adapting for 1 day, the vestibulo-ocular reflex (VOR) to yaw rotation was partially restored (Gong and Merfeld, 2002). Furthermore, a prosthetic semicircular canal was implanted in squirrel monkeys with plugged lateral SCCs. Despite initially having low VOR gain, between 1 or 2 weeks after implantation, the VOR gain to yaw rotation gradually recovered. With chronic stimulation, the gain increased, the rotational axis improved, and the VOR became symmetric. These results confirmed that brain plasticity changes might occur, producing an adaptive VOR gain, axis, and symmetry modification when provided with chronic motion-modulated electrical stimulation (Lewis et al., 2010). Using the prosthetic device also demonstrated that chronic stimulation, patterned as a function of head movement, can provide adequate information for the brain to generate a VOR. Additional research demonstrated that using a prosthetic device that modulates electrical stimulation according to head movements, used for several months, increased the gain and symmetry of the VOR, most likely due to plastic changes in the brain that contributed to the functionality of the prosthetic device. These studies in animal models provided the evidence that defined the feasibility of vestibular prostheses (Lewis et al., 2002/2003; Wall et al., 2002/2003; Merfeld et al., 2006; Merfeld et al., 2007; Lewis et al., 2010).

An advanced prosthetic prototype consisting of various electrodes, one applied to each semicircular canal (Della Santina et al., 2005, 2007, 2016), was further improved with a decrease in size and power consumption, a new microelectrode array design with multiple current sources, and circuitry for *in vivo* measurement of electrode impedances (Chiang et al., 2011). Tests in bilaterally vestibular-deficient rodents and rhesus monkeys showed that the Multichannel Vestibular Prosthesis could partially restore the three-dimensional vestibulo-ocular reflex (VOR) (Fridman and Della Santina, 2012). Since most research has been done with unilateral implants to encode bidirectional motion, stimulation artificially elevated the vestibular afferent neurons’ basal action potential discharge rate. Then, the prosthetic device modulated the pulse rate around this increased baseline, allowing for coding accelerations in the excitatory and inhibitory directions of the respective semicircular canal. Otherwise, the bidirectional coding of head movements naturally achieved by the joint activity of the left and right SCC would be lost (Fridman and Della Santina, 2013; Della Santina et al., 2016; Fridman, 2017; Aplin and Fridman, 2019). The influence of stimulus characteristics on the VOR was studied by varying biphasic current pulse frequency, amplitude, duration, and the interphase gap (the time between the cathodic and anodic current in a biphasic current balanced pulse). Results showed that pulse frequency modulation of short biphasic pulses should be the basis for future optimization of stimulus protocols. Although eye alignment is better with pulse-rate-modulated stimuli, magnitude responses are smaller than when using amplitude-modulated stimuli (Davidovics et al., 2011).

Most recent vestibular implant (VI) prototypes use a frequency modulation-encoding scheme, as it is used in cochlear implants. Pulse timing errors caused by temporal discretization are inherent to this system’s implementation. Therefore, electrically evoked VOR were studied in two rhesus macaques using: (i) a smooth pulse frequency modulation map; (ii) a discretized map corrupted by temporal errors. The introduction of pulse timing errors produced negligible effects on responses across all canals, indicating that pulse timing errors did not degrade the performance of the potentially combined cochlear and VI (Boutros et al., 2019b).

The low velocity of the VOR to head movements and misalignment between the direction of head motion and of prosthetically elicited VOR were two significant problems, although, as previously noted, misalignment and gain tend to be corrected by plastic adaptation (Davidovics et al., 2013). Results also showed that head movements elicited by horizontal SCC stimulation significantly contributed to gaze stabilization, complementing the VOR (Mitchell et al., 2013). It has also been shown in two rhesus monkeys that head orientation perception improves using a VI that stimulated the three SCC can improve graviception, indicating that the brain may extract information about the head’s orientation from the SCC activity (Karmali et al., 2021).

The most advanced version of the multichannel vestibular prosthesis (MVP-2a) was designed based on an application-specific integrated circuit (ASIC) which included a multiplicity of electrodes (sixteen) in a highly integrated system (Hageman et al., 2016). Tests of this device in an implanted monkey showed that eye movements in the expected plane with gains from 0.04 to 0.33 were obtained. The performance of this new device was like that previously reported with larger devices, with the advantage of having a much smaller, implantable device and lower power consumption. Recent developments have also used ASICs with more complex mathematical processing (Töreyin and Bhatti, 2016).

## Human implantation studies

Pioneering work in experimental human implantation was performed on three subjects who underwent cochlear implant surgery. The subjects had pre-existing spontaneous and positional horizontal nystagmus, their posterior ampullary nerve was surgically exposed, and eye movement was recorded during electrical stimulation (Wall et al., 2007). These works proved that vestibular SCC stimulation in humans is feasible: the nystagmus slow component velocity can be modulated by the pulse rate (maximum rate of 200 pulses/s) and by the stimulus amplitude with a response threshold of 200 μA. The eye movements were mainly vertical, and no interaction was found with ongoing horizontal eye movements (Wall et al., 2007). A subsequent experimental series showed that the lateral canal nerve stimulus elicited horizontal eye movements (Guyot et al., 2011a). Stimulation in the posterior ampullary nerve showed that successive stimulation cycles resulted in progressively shorter duration nystagmus, and once adapted, constant stimulation produced smooth conjugated eye movements, showing that human subjects may adapt to continuous electrical stimulation of the vestibular system (Guyot et al., 2011b). Adaptation is particularly relevant since it shows that plastic changes occur at central neurons, implying that plasticity will be preserved in cases of severe sensorineural BVD (Mitchell et al., 2017).

A device using a pacemaker (UW/Nucleus Vestibular Implant) based on a modified Nucleus Freedom Cochlear Implant with motion-modulated output demonstrated good preservation of auditory and vestibular function in animal models; however, its use in patients caused a reduction in auditory function, attributed to various complications most probably due to surgical technique which implies opening the bone labyrinth using noisy drills, apart from the fact that bone opening may mechanically destabilize the inner ear. The electrode insertion may degrade the endolymph to perilymphatic isolation.

Vestibular function showed a postoperative loss in the implanted ear of all subjects (Phillips et al., 2013; 2015a). Moreover, a combined version of the Nucleus Freedom implanted in 3 human subjects, with two stimulation sites on the SCC and 16 stimulation sites for intracochlear stimulation, showed the adequate independent performance to elicit eye movements or sound sensation alone. However, combined stimulation produced higher eye velocities and an increment in the perceived pitch and loudness, presumably because of the activity overlap between the auditory and the vestibular sensory organs. These findings highlight another critical challenge yet to be overcome: the vestibular-auditory interaction of the implanted device (Phillips et al., 2020).

Various experimental series with human implantation have followed using a cochleo VI prototype coupled to a three-axis gyroscope (Nguyen et al., 2014), providing the first evidence of restoration of an artificial VOR in patients (Perez-Fornos et al., 2014; Guinand et al., 2015; Guyot and Perez-Fornos, 2019). The prototype electrodes were implanted on the ampullary nerves of the three SCC, though only one vestibular electrode at a time was activated; the active electrode (either in lateral, posterior, or anterior SCC) varied depending on the experiment. Currently, visualization techniques that guarantee a more precise positioning of the electrode tip in the center of the SCC during implantation are being explored (Stultiens et al., 2020).

The VOR analysis of implanted subjects using sinusoidal stimulation instead of square biphasic pulses showed that the total peak eye velocity of the VOR had a significant frequency dependence. In contrast, the angle, habituation index, and asymmetry of the VOR showed no significant frequency-dependent effect. The frequency dependence of the VOR peak velocity in the VI mimicked the frequency dependence of the VOR in non-implanted normal subjects (van de Berg et al., 2015).

Analyzing the influence of pulse modulation strategy to encode head rotation has become increasingly necessary with modified cochlear implants for vestibular stimulation (Valentin et al., 2013; Golub et al., 2014; Perez-Fornos et al., 2014; Guinand et al., 2015). The efficacy of modulating the stimulus pulse rate or the pulse amplitude suggests exploring a combined strategy. While pulse amplitude modulation evokes larger eye movements, eye alignment was better with pulse rate modulation (Nguyen et al., 2016). In addition, pulse amplitude modulation consistently caused a more significant ensemble-firing rate of neurons, which coincides with a larger magnitude of eye movement produced by amplitude modulation of the stimulus (Nguyen et al., 2016). Phase duration of pulses, pulse baseline rate, and pulse amplitude are the main parameters under investigation for defining the ideal operation of a VI. Using the Geneva-Maastricht VI prototype, the current intensity modulation was the factor that had the most significant impact on the peak velocity of the VOR (Crétallaz et al., 2020).

A latent problem for patients with VI is that the residual vestibular function might interact with the implant input, with unknown consequences (Porciuncula et al., 2012). This problem was addressed in four bilateral vestibulopathy patients implanted with the Geneva-Maastricht vestibular prosthesis (van de Berg et al., 2017a). The stimulus amplitude was set in the middle of the dynamic range measured for each patient, using supraphysiological baseline stimuli, generating bidirectional vestibular sensations with a unilateral prosthesis (van de Berg et al., 2011). However, the residual activity of a semicircular canal did not counteract the misalignment of the implant input (van de Berg et al., 2017a). These results add to the complexity of signal processing required, in this case, to cope with residual vestibular function. It is worth noting that stimulation was performed acutely in this study, and no long-term adaptation to the prosthetic device was allowed. Adaptation will probably help to cope with misalignments and integrate the implant input with remnant vestibular function. In fact, among the selection criteria proposed for VI in humans is that BVD is peripheral, emphasizing the role of residual vestibular function interacting with the prosthetic devices (van de Berg et al., 2020).

Further tests analyzed the vestibular prototype’s capability to improve gaze stabilization and anomalous visual acuity in dynamic conditions (Guinand et al., 2016). For example, by testing the dynamic visual acuity using letters displayed on a computer monitor while the subject walked on a treadmill, activating the prosthetic device provided motion information and restored visual acuity in the dynamic condition. As noted by the authors, “This was the first demonstration of functional rehabilitation using motion-modulated electrical stimulation of the vestibular nerve in humans” (Guinand et al., 2016). Additionally, the vHIT was used to determine more complex and dynamic responses in patients implanted unilaterally and the lateral or anterior canals stimulated. The vHIT studies demonstrated that the implant might restore VOR in a motion-modulated form. In most cases, the gain for excitatory head impulses was more significant than for inhibitory impulses, although a high intra- and inter-subject variability was reported (Guinand et al., 2017). Similarly, a case study of an implanted patient pointed to a high-frequency dynamic visual acuity restoration with positive stimulation of the lateral semicircular canal and baseline stimulation using the functional HIT, a variant of the vHIT (Starkov et al., 2020).

Due to previous reports concerning auditory function decrease in patients with VI (Phillips et al., 2015b), and given that the Geneva–Maastricht group has exclusively implanted patients who were deaf in the implanted ear (Perez-Fornos et al., 2014; Guyot and Perez-Fornos, 2019), it is still necessary to define the degree of hearing loss produced after VI. Despite the encouraging results in animal models (Bierer et al., 2012; Rubinstein et al., 2012), there is still insufficient data to ensure the preservation of auditory function in humans receiving a VI. Experts conjecture about the differences between the two species, such as particular anatomic features and recovery capacity. The state of the auditory-vestibular system (healthy in animal models, with disease in humans) before implantation also appears critical (Golub et al., 2014). A case report analyzing auditory function during a trans labyrinthine surgery showed no significant reduction of auditory brainstem response during the insertion and removing the electrodes in the lateral and the posterior SCC (van de Berg et al., 2017b). Recordings of compound action potential from both cochlear and vestibular electrode setups in four human subjects demonstrated that VI provides information on the efficacy of electrode implantation and objective measurement of the effect of electrode activation current spread (Nguyen et al., 2017).

A device derived from a Nucleus CI24M cochlear implant with bidirectional telemetry and with three active electrodes for vestibular stimulation, were implanted unilaterally in patients selected following Barany criteria for VI, with an absence of vestibular evoked myogenic potentials (VEMPs) and profound deafness (Ramos de Miguel et al., 2017). Subjects were implanted with an extralabyrinthine electrode in the inferior vestibular nerve in a region mainly containing afferent neurons from the saccular macula and likely from the posterior SCC. The device produced a constant rate (no mechanical modulation) stimulation with biphasic pulses (25 μs per phase) and a frequency of 900 or 1,200 pulses per second (Curthoys et al., 2022). In all cases, activation of the electrodes produced ocular VEMPs responses due to otolithic activation. A set of five patients implanted with this device (of a total of ten implanted by January 2022) were studied using various vestibular tests, including the Dizziness Handicap Inventory (DHI), Dynamic Gait Index (DGI), vestibular evoked myogenic potential (VEMPS), Subjective Visual Vertical (SVV), and posturography. In all cases, these vestibular tests significantly improved (Ramos Macias et al., 2020). The authors also compared the visual acuity in static and walking conditions, oscillopsia severity questionnaire (OSQ), and vHIT, before and after activation of the VI. Results show a significant improvement in dynamic visual acuity, OSQ score, and the expression of organized corrective saccades with no changes in gain in the vHIT test. The authors conclude that the otolithic VI significantly restores dynamic visual acuity (Rodríguez-Montesdeoca et al., 2022). Ramos de Miguel states, “The otolith system must be considered superior to the SCC system as illustrated by evolution, clinical evidence, and physical principles” (Ramos de Miguel et al., 2020). The authors have developed a European Project denominated BionicVEST to share and expand the use of the technology; they propose using the otolithic system as a primary target for VI. The remarkable effect of this “relatively simple device” has led to the proposal of a “saccular substitution hypothesis,” establishing that neural activity produced at the central nervous system is essential for cerebellar, basal ganglia, and mesencephalic motor control regions to organize the motor behavior, and that constant rate saccular nerve stimulation is substituting for that neuronal activity in a form similar to deep brain stimulation (Curthoys et al., 2022).

Most recently, four patients with bilateral vestibular loss were unilaterally implanted with the Labyrinth Devices Multichannel Vestibular Implant System (LD-MVI), essentially based on the widely studied Della Santina proposal (Della Santina et al., 2007). Results using the LD-MVI showed that, after an adaptation period, continuous use of the device produced stable VOR responses aligned approximately with the specified 3D head rotation axis (Boutros et al., 2019a). LD-MVI has now been implanted in eight subjects, and locomotor tests performed after 1 year of use of the VI show a significant improvement in posture, gait, and, most significantly, quality of life (Chow et al., 2021). This work is the first report of a successful mechanically modulated VI device that allowed functional recovery, along with the BionicVest represents a significant leap in developing the VI (Table 1).

**TABLE 1:** Vestibular prosthesis comparison.

	VI UW/Nucleus freedom	VI Geneva-Maastricht	LD-MVI	Bionic vest
Sensor	None	3D-gyroscope	3D-gyroscope	None
Processing and coding unit	Pacemaker fixed pulse rate	Linear transfer function to modulate pulse amplitude and frequency	Codes for amplitude and rate of pulse frequency with a 32-point piecewise-linear approximation to a sigmoid curve	Pacemaker fixed pulse rate and amplitude
Electrode implantation	Perilymphatic space adjacent to each SCC ampullae	Inserted in the three SCC ampullae	Inserted in the three SCC ampullae	Adjacent to the saccular nerve
Behavioral response	Modulation of ocular movements Auditory and vestibular loss No further use.	VOR, vestibulo-colic, and vestibulo-espinal reflex restoration. Cognitive response	VOR restoration, Gait, position, and stabilization. Significant improvement in the quality of life.	Gait and gaze stabilization. Significant improvement in the quality of life.

## Conclusion

It took about 30 years from the first studies about vestibular prosthesis feasibility in the early 90 s to reach successful human implantation. The pioneering basic research studies that analyzed the responses of electrical stimulation of vestibular nerves in animal models (Cohen et al., 1966; Suzuki et al., 1969; Goldberg et al., 1984) along with the coincident technological advances in miniaturization of various sensors and processors, including gyroscopes and accelerometers, allowed researchers to envisage the feasibility of developing an artificial vestibule (Shkel, 2001; Shkel and Zeng, 2006). At the turn of the millennium, Gong and Merfeld (2000) made the first proposal for an artificial vestibular prototype. This work marks the beginning of the development of vestibular prostheses; initially, a single SCC prototype was envisioned, and other proposals were quickly developed, including three-dimensional sensors and systems with electrodes applied to all the SCCs (Della Santina et al., 2005, 2007). These prototypes were tested in various animal models, demonstrating the ability to produce the VOR in animals with deep vestibular hypofunction after drug treatment or by acute occlusion of the SCC. This evidence gave rise to the first implants in patients. However, VI in humans has produced hearing loss as a side effect (Phillips et al., 2015b), although in animal models, VI is accomplished without an apparent loss of auditory function.

It is noticeable that the evaluation of prosthetic systems is performed using the vestibulo-ocular reflex (VOR) as the standard to determine the re-establishment of vestibular function (Guinand et al., 2017). Recently, electrically evoked VOR allowed the eye movement assessment in eight implanted subjects by considering its asymmetrical nature (Starkov et al., 2022). However, subjects with vestibular damage have complex spatial navigation problems that have not been addressed in human VI studies except in one series implanted with the Geneva-Maastricht VI (Perez-Fornos et al., 2019). Most of the studies analyze the VOR, which measures the activation of SCC in a parametric form. In addition, it is essential to consider the postural responses related to maintaining static and dynamic equilibrium and cognitive processes that are fundamental elements of locomotor behavior in humans (Hitier et al., 2014). A systematic review of the biomechanical outcome of invasive and non-invasive electrical vestibular stimulation, including 19 Q1 and 2 Q2 research papers, found just one publication on postural evaluation after VI (Haxby et al., 2020).

Data from six participants 1 year after implantation showed that all subjects presented a reduced hearing function (Chow et al., 2021). However, most patients will select losing auditory function in the implanted ear if they gain postural and gaze stability. Additionally, it seems essential to consider complex cognitive processes related to navigation and the body scheme to evaluate the implant function (Lopez, 2013).

Given the collateral damage to hearing observed in some of the implanted patients and because other implants have been done in patients with profound deafness (Guyot and Perez-Fornos, 2019), it is possible to conclude that, to date, the indication for the vestibular implant must be limited to patients with unilateral deafness. In the future, electrode improvement, stimulation selectivity, surgical techniques (Handler et al., 2017; van de Berg et al., 2017b; Schier et al., 2018), and better methods of electrode fixation will surely expand the possibility of vestibular implantation in subjects, producing no or mild auditory damage.

Although otolithic organs have a complex structure, which unlike the SCC produce multidimensional responses, they provide the subject with information that has an essential influence on the position of the head and its orientation to the normal, which is essential to avoid falls (Ramos de Miguel et al., 2020). Implantation exclusively in SCC produces a situation analogous to that found in microgravity, in which the otolithic inputs are altered or absent, but the SCC remains functional. This situation seems to induce a neurosensory conflict that causes plastic changes leading to a reduction of the gain of the afferent input from the SCC with critical functional consequences (Kornilova and Kozlovskaya, 2003; Kornilova et al., 2017). Although, in monkeys with VI in the three SCC the brain may extract information about head position (Karmali et al., 2021). Also in humans wearing the Geneva-Maastricht VI, acoustically elicited cervical vestibular evoked myogenic potentials (cVEMPs) can be elicited, suggesting that VI in SCC can improve postural control (Perez-Fornos et al., 2019).

From a technical standpoint, prototype implants’ sensors, power, and miniaturization are already solved. However, developing suitable stimulation regimes that reflect the complexity of the subject’s displacement in their environment and the set of accelerations to which the head is naturally exposed is a field of significant growth and interest. It is necessary to develop protocols to produce complex patterns of stimulation that consider the modulation of frequency, amplitude, and waveform, as well as, eventually, enable contextualization according to the subject’s movement and adapt the operation of the prosthetic device to the behavioral context.

An aspect to consider in developing vestibular prostheses, whether implanted or non-invasive, is the potential conflict in differentiating self-generated movements from passive movements. Furthermore, the activity of the efferent system must not be disregarded, as it has a relevant participation by contributing to exafference (Angelaki and Cullen, 2008; Cullen, 2012; Laurens and Angelaki, 2017). An open question remains about how the efferent system may interact with an input that will not receive modulatory feedback from the CNS.

In conclusion, a lot has been done in developing a vestibular implant, although we still do not think the vestibular implant methodology is solved. An exciting development has been achieved by adding an otolithic organ-stimulating electrode. However, there are still no studies of a device combining SCC and otolithic organ stimulation in humans. Most probably, advances in the surgical procedures for implantation and improvement in the electrical stimulating patterns will improve the properties of the VI and help to avoid the hearing loss reported in nearly all VI recipients. Together with the development of modern methods of vestibular rehabilitation, the use of vestibular implants will undoubtedly improve the therapeutic alternatives offered to patients with vestibular damage.

## Author contributions

ES conceived the review. ES, AP, and RV contributed similarly by reviewing the literature and writing the manuscript. All authors contributed to the article and approved the submitted version.
